# Double-edged role of radiotherapy in patients with pulmonary large-cell neuroendocrine carcinoma

**DOI:** 10.7150/jca.32446

**Published:** 2019-10-19

**Authors:** Yuanzhu Jiang, Cong Lei, Xufeng Zhang, Yangang Cui, Keying Che, Hongchang Shen

**Affiliations:** 1 Department of Thoracic Surgery, Shandong Provincial Hospital Affiliated to Shandong University, 324 Jingwu Road, Jinan, 250021, P.R. China; 2 Department of Oncology, Shandong Provincial Hospital Affiliated to Shandong University, 324 Jingwu Road, Jinan, 250021, P.R. China; 3 Institute of Oncology, Shandong Provincial Hospital Affiliated to Shandong University, 324 Jingwu Road, Jinan, 250021, P.R. China; 4 Department of kidney Transplantation, Second Hospital of Shandong University, 247 Beiyuan Road, Jinan, 250000, P.R. China

**Keywords:** pulmonary large-cell neuroendocrine carcinoma, surgery, radiotherapy, SEER

## Abstract

**Purpose:** Pulmonary large-cell neuroendocrine carcinoma (LCNEC) is classified as non-small-cell lung cancer, but has characteristics similar to small-cell lung cancer. This study was performed to evaluate the effect of surgery and radiotherapy on patients with LCNEC.

**Materials and Methods:** We analyzed 1,619 patients with stage I-III LCNEC, identified from the Surveillance, Epidemiology, and End Results database, diagnosed from 2000 to 2013. The Kaplan-Meier analysis and the Cox proportional hazard model were used to study patient prognosis.

**Results:** Overall, 869 (53.7%) stage I LCNEC patients, 203 (12.5%) stage II patients, and 547 (33.8%) stage III patients were included in the analysis. Various surgery types were all associated with higher overall survival (OS) and lung cancer-specific survival (LCSS) than no surgery, with the following HRs: 0.334 (OS) and 0.279 (LCSS) for lobectomy, 0.468 (OS) and 0.416 (LCSS) for partial/wedge/segmental resection, and 0.593 (OS) and 0.522 (LCSS) for pneumonectomy (all p < 0.05). OS and LCSS of stage I and II LCNEC patients were not improved by radiotherapy (stage I: OS p = 0.719, LCSS p = 0.557; stage II: OS p = 0.136, LCSS p = 0.697). However, in stage III patients, radiotherapy significantly improved both OS and LCSS (p < 0.001). Following multivariate analysis, increased age, male patients, radiotherapy and diagnosed at stage II or III were all independent risk factors for LCNEC (all p < 0.05).

**Conclusion:** Lobectomy had the best outcome for OS and LCSS in stage I-II LCNEC. For stage III LCNEC patients, radiotherapy can significantly improve survival time. However, in LCNEC patients undergoing surgery, radiotherapy may reduce survival time.

## Introduction

Pulmonary neuroendocrine tumors originate from the endocrine cells of the lung and bronchial epithelium, accounting for 20% of primary lung cancers. Among them, pulmonary large-cell neuroendocrine carcinoma (LCNEC) accounts for 3% 1, 2. LCNEC is pathologically classified as non-small-cell lung cancer (NSCLC), being regarded as a high-grade neuroendocrine tumor 3, 4. However, LCNEC shares several clinico-pathological characteristics with small-cell lung cancer (SCLC), including high degree of malignancy, poor patient prognosis, smoking-related disease and common neuroendocrine gene expression, which has attracted the attention of numerous scholars 5-7.

The diagnosis of LCNEC requires assessing both morphology and neuroendocrine differentiation by IHC 8-10. In the current WHO classification, some of the features used to classify a tumor as LCNEC overlap with those applied for SCLC, NSCLC, and carcinoids 11. As LCNEC is a very rare disease, difficult to diagnose and treat, only few data are available, and clinical trials are difficult to be performed. To date, optimal clinical management has not been established 12, 13. Surgery is generally the first choice for early-stage patients 14, as several studies have reported that LCNEC patients can benefit from early surgical interventions, achieving satisfactory results 15-18. However, many people with pulmonary LCNEC have a high incidence of recurrence after surgery, even when performed for early-stage disease. There is evidence that chemotherapy is an effective method for preventing disease relapse 19-21. Moreover, molecular subtypes of pulmonary LCNEC can predict the outcome of chemotherapy 22. Nevertheless, data on radiotherapy in pulmonary LCNEC patients are limited 23, 24. The role of radiotherapy in LCNEC remains unclear, and further research is necessary to establish its effectiveness 12.

To further investigate the influence of surgery and radiotherapy on prognosis and, in particular, to identify the optimal therapeutic method for patients with pulmonary LCNEC, we acquired data from the Surveillance, Epidemiology, and End Results (SEER) database and performed a retrospective analysis.

## Materials and Methods

### Data Source and Ethical Regulations

The SEER database is a National Cancer Institute (NCI) program, which encompasses information about cancer incidence and patient survival in the United States (SEER website [www.seer.cancer.gov]). Comprehensive data of diagnosed patients are included in the NCI SEER 18 Registries (SEER*Stat Database: Incidence—SEER 18 Regs Research Data + Hurricane Katrina Impacted Louisiana Cases, Nov 2015 Sub). The Institutional Review Board (IRB) approve this study. And an ethics committee is not required because individual patient data are de-identified.

### Study Population

Our study sample consisted of 1,619 patients, with exfoliative cytologically and histologically confirmed American Joint Committee on Cancer (AJCC) stage I-Ⅲ LCNEC, diagnosed from 2000 to 2013. The histology codes were identified according to International Classification of Diseases for Oncology (3rd Edition, ICD-O-3, 8013). Included patients were from SEER code 8013/3 (large-cell neuroendocrine carcinoma), and morphology site “lung and bronchus”. Patients with undefined nodal status or M1 disease were excluded. In addition, we also excluded patients with unknown race, tumor size, surgery/surgery type, or survival months. Data of interest on therapeutic methods included type of surgery and radiotherapy administration. Chemotherapy details were not available. We assumed that almost all these patients received systemic therapy.

### Statistical Analyses

The primary outcomes of this study were overall survival (OS) and lung cancer-specific survival (LCSS). OS and LCSS were analyzed using Cox regression. Kaplan-Meier analysis with the log-rank test was used to estimate OS and LCSS, and generate survival curves to compare patients who underwent surgery or received radiotherapy with those who did not 25. Univariate analysis and multivariate analyses were carried out by the Cox proportional hazard model. Moreover, the multivariate Cox regression analysis included covariates that were significant in the univariate analysis, to determine which factors significantly influenced survival. A two-sided P-value < 0.05 was considered statistically significant. All data were analyzed using the SPSS software package, version 20.0 (IBM, SPSS Statistics, Chicago, IL).

## Results

### Patient characteristics

We identified 1,619 patients with stage I-III LCNEC, diagnosed from 2000 to 2013. Patients' baseline demographic characteristics are presented in Table 1. There were 770 (47.6%) female and 849 (52.4%) male patients, with a median age of 67 years (18-94). Most patients were Caucasian (85.3%) and married (85.7%). Regarding treatment, 1020 patients underwent surgery only, 65 patients underwent radiotherapy only, 167 patients underwent surgery + radiotherapy, 138 those who had postoperative radiation therapy (PORT) and 367 those without such treatment. Of the patients who underwent lung resection, 850 (52.5%) underwent lobectomy, 278 (17.2%) underwent partial/wedge/segmental resection, and 56 (3.4%) underwent pneumonectomy. Patients with stageⅠLCNEC accounted for 53.7% of the total, stage II for 12.5%, and stage III for 33.8%.

### Effect of surgery on survival

**Figure 1:**
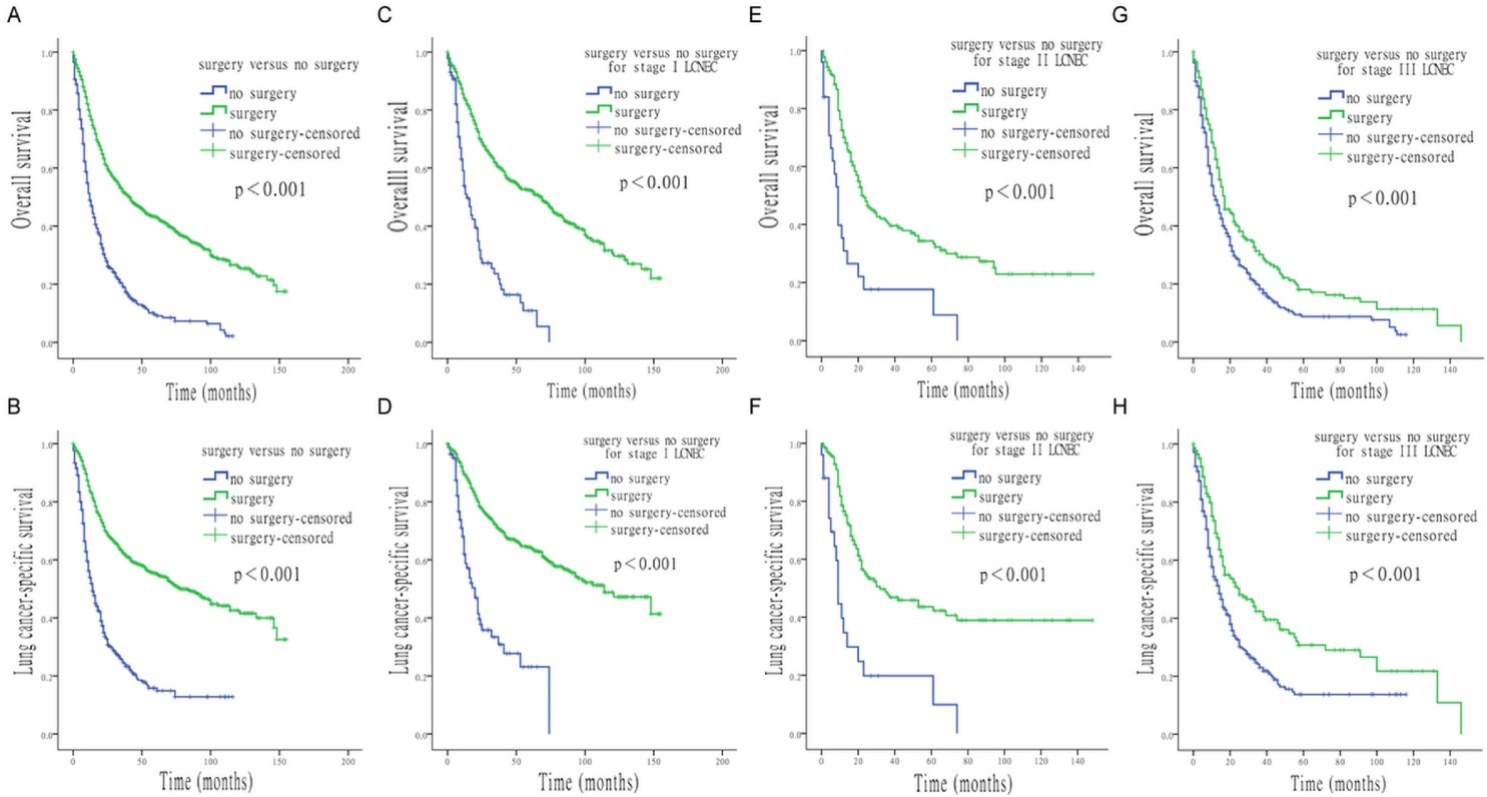
** Survival analysis for OS and LCSS based on surgery or no-surgery, at each stage of LCNEC**. (A) OS of patients treated with or without surgery. (B) LCSS of patients treated with or without surgery. (C) OS of stage I LCNEC patients, treated with or without surgery. (D) LCSS of stage I LCNEC patients, treated with or without surgery. (E) OS of stage II LCNEC patients, treated with or without surgery. (F) LCSS of stage II LCNEC patients, treated with or without surgery. (G) OS of stage III LCNEC patients, treated with or without surgery. (H) LCSS of stage III LCNEC patients, treated with or without surgery.

Results of the univariate Cox analysis of OS and LCSS are shown in Table 2. Survival analysis revealed that age, gender, surgery vs. no-surgery, and tumor-node-metastasis (TNM) stage were all significant factors for OS and LCSS. Survival time was significantly improved for patients with stage Ⅰ to Ⅲ LCNEC when undergoing surgical interventions (all p < 0.001; Figure 1). For OS, median survival time for all LCENC patients treated with surgery was 41.0 months (95% CI, 34.9 to 47.1 months), which was significantly longer than that of patients who did not undergo surgery (12.0 months; 95% CI, 10.3 to 13.7 months). The median overall survival (OS) for surgically and non-surgically treated LCNEC patients was 69.0 months (95% CI, 57.7 to 80.3 months) and 14.0 months (95% CI, 9.7 to 18.3 months) in stage I, 23.0 months (95% CI, 16.8 to 29.2 months) and 9.0 months (95% CI, 6.7 to 11.3) in stage II, and 17.0 months (95% CI, 13.4 to 20.6 months) and 12.0 months (95% CI, 9.9 to 14.1 months) in stage III, respectively. For LCSS, median survival time for all LCENC patients treated with surgery was 78.0 months (95% CI, 63.5 to 92.5 months), which was also significantly longer than that of patients without surgery (14.0 months; 95% CI, 11.7 to 16.3 months). The median LCSS survival for surgically and non-surgically treated patients was 114.0 months (95% CI, 88.2 to 139.8 months) 20.0 months (95% CI, 13.8 to 26.2 months) in stage I, 32.0 months (95%CI, 11.2 to 52.8 months) and 9.0 months (95%CI, 7.6 to 10.4 months) in stage II, and 25.0 months (95%CI, 15.8 to 34.2 months) and 14.0 months (95%CI, 11.5 to 16.5 months) in stage III, respectively.

**Figure 2:**
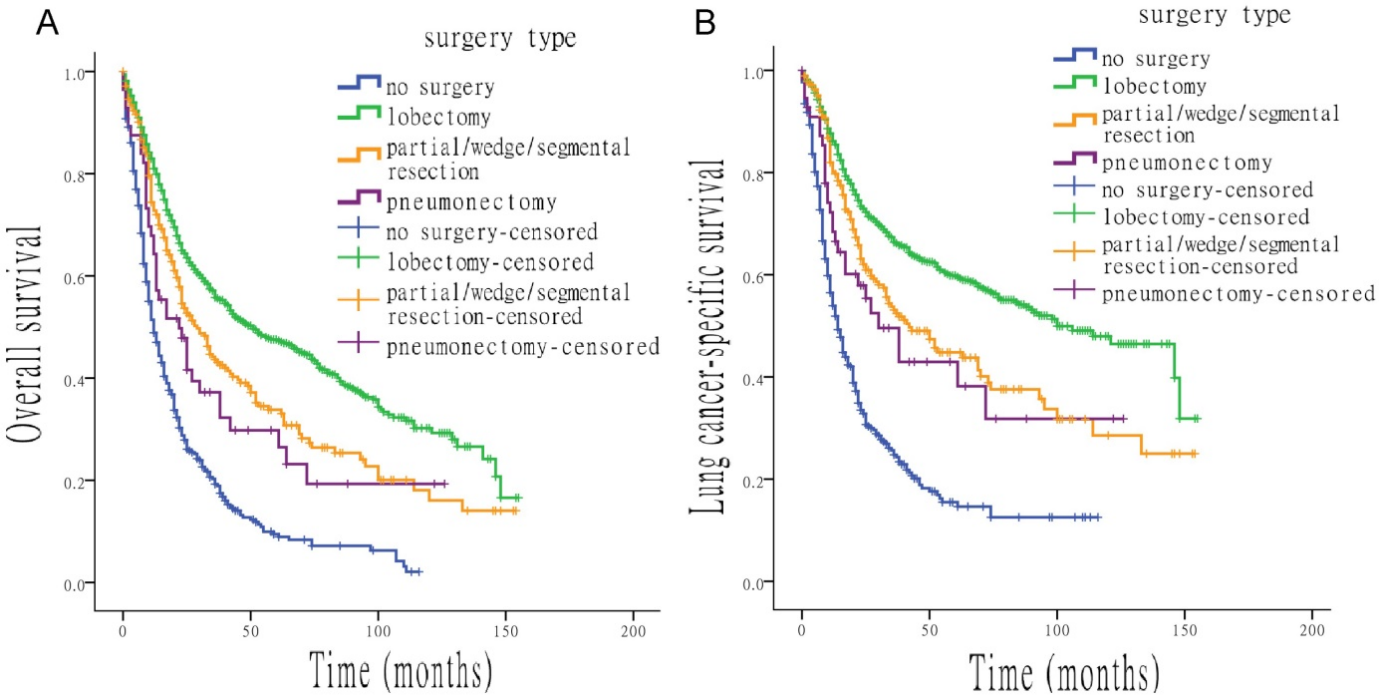
** Survival analysis for OS and LCSS based on type of surgery.** (A) OS of all patients, treated with different types of surgery. (B) LCSS of all patients, treated with different types of surgery.

Various types of surgery were all associated with higher OS and LCSS than no surgery, with the following HRs: 0.334 (OS) and 0.279 (LCSS) for lobectomy, 0.468 (OS) and 0.416 (LCSS) for partial/wedge/segmental resection and 0.593 (OS) and 0.522 (LCSS) for pneumonectomy (all p < 0.05). Figure 2 shows the effect of multiple surgical interventions on survival. For OS, people who underwent lobectomy had the highest median survival time, of 51.0 months (95% CI, 39.1 to 62.9 months), which was significant longer than that of patients without surgery (12.0 months; 95% CI, 10.2 to 13.8 months). Median overall survival time of patients who underwent partial/wedge/segmental resection was 29.0 months (95% CI, 23.2 to 34.8 months), compared with pneumonectomy was 22.0 months (95% CI, 11.5 to 32.5 months). For LCSS, people with lobectomy also had the best median survival, of 100.0 months (95% CI, 77.0 to 123.0 months), whereas the median survival of patients without surgery was only 14.0 months (95% CI, 11.8 to 16.2 months). Median survival time of patients who underwent partial/wedge/segmental resection and pneumonectomy was 42.0 months (95% CI, 28.8 to 55.2 months) and 30.0 months (95% CI, 14.8 to 45.2 months), respectively.

### Effect of radiotherapy on survival

**Figure 3:**
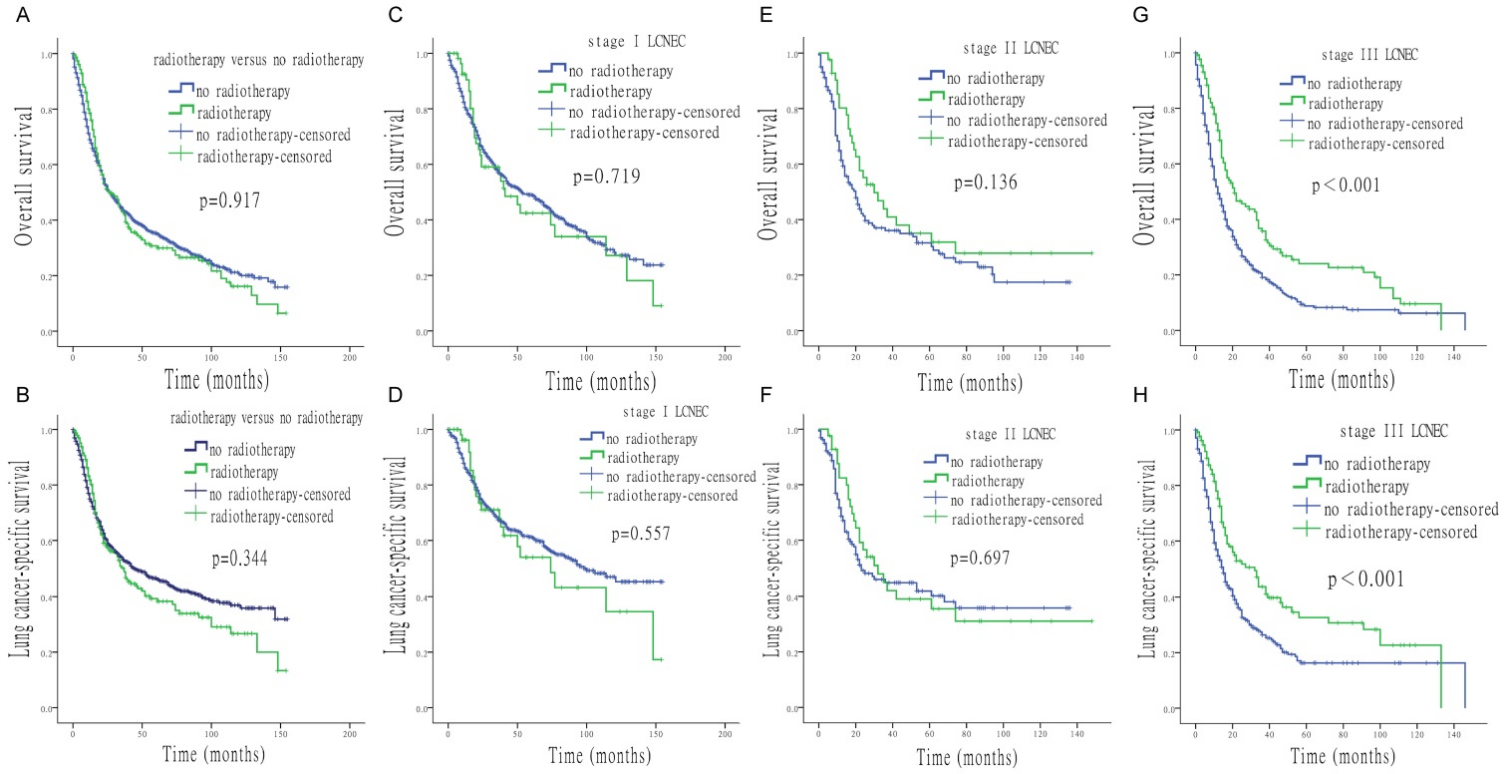
**Survival analysis for OS and LCSS based on radiotherapy or no-radiotherapy, at each stage of LCNEC.** (A) OS of patients treated with or without radiotherapy. (B) LCSS of patients treated with or without radiotherapy. (C) OS of stage I LCNEC patients, treated with or without radiotherapy. (D) LCSS of stage I LCNEC patients, treated with or without radiotherapy. (E) OS of stage II LCNEC patients, treated with or without radiotherapy. (F) LCSS of stage II LCNEC patients, treated with or without radiotherapy. (G) OS of stage III LCNEC patients, treated with or without radiotherapy. (H) LCSS of stage III LCNEC patients, treated with or without radiotherapy.

Figure 3 shows that OS and LCSS of stage Ⅰ and Ⅱ LCNEC patients were not improved by radiotherapy (stage Ⅰ: OS p = 0.719, LCSS p = 0.557; stage Ⅱ: OS p = 0.136, LCSS p = 0.697). However, in stage Ⅲ patients, radiotherapy significantly improved both OS and LCSS (p < 0.001).

**Figure 4:**
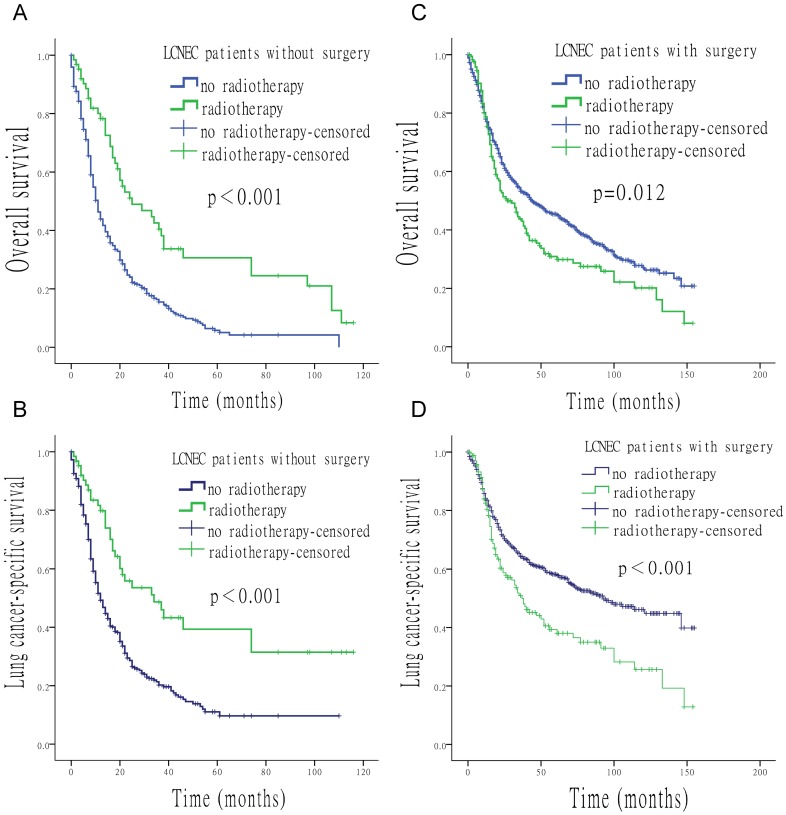
** Survival analysis for OS and LCSS relative to surgery and radiotherapy.** (A) OS of patients who did not undergo surgery, treated with or without radiotherapy. (B) LCSS of patients who did not undergo surgery, treated with or without radiotherapy. (C) OS of patients who underwent surgery, with the addition or in the absence of radiotherapy. (D) LCSS of patients underwent surgery, with the addition or in the absence of radiotherapy.

**Figure 5:**
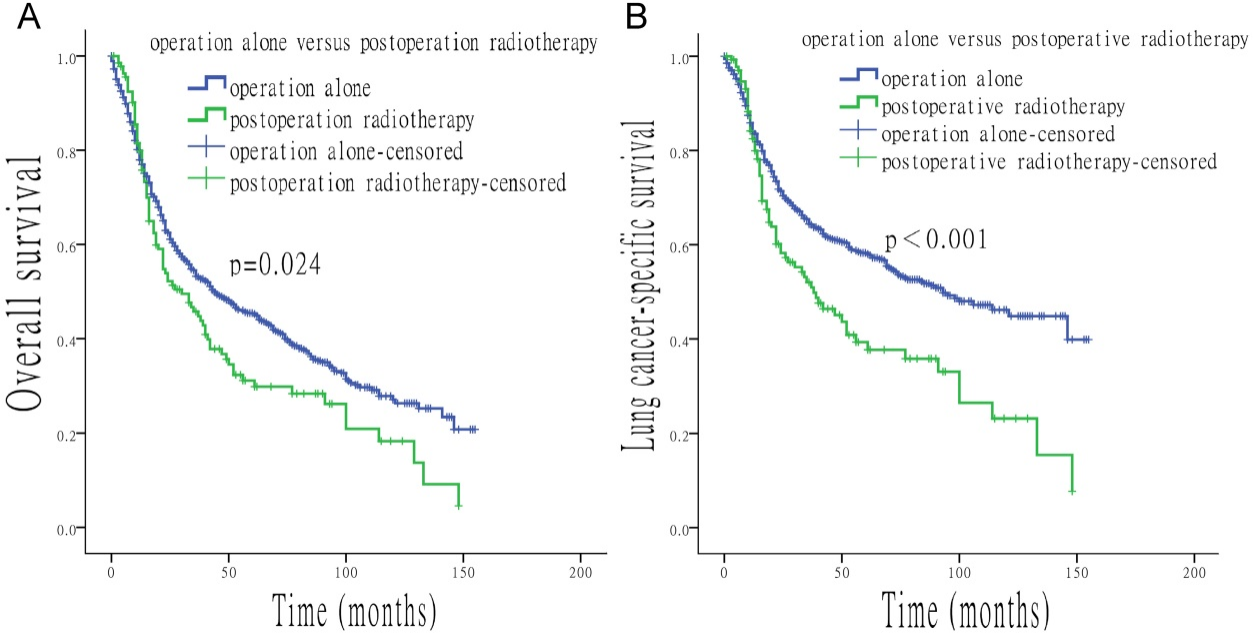
Survival analysis based on surgery alone, or with the addition of postoperative radiotherapy. (A) OS of patients treated with surgery alone, or with surgery and postoperative radiotherapy. (B) LCSS of patients treated with surgery alone, or with surgery and postoperative radiotherapy.

When surgery was performed, the use of radiation was associated with a shorter median survival time (OS: 27 *vs*. 44 months, p = 0.012; LCSS: 37 *vs*. 93 months, p < 0.001). In contrast, longer median survival time was found with the use of radiation when surgery was not performed (OS: 25 *vs*. 11 months, p < 0.001; LCSS: 34 *vs*. 12 months, p < 0.001) (Figure 4). Compared to patients who underwent postoperative radiotherapy, patients with surgery alone had a longer survival time (OS: 44 *vs*. 30 months, p = 0.024; LCSS: 93 *vs*. 38 months, p < 0.001) (Figure 5).

### Multivariate analysis on survival

Table 3 shows that increased age, male patients, radiotherapy, and stage II or III at diagnosis, were all significant risk factors for LCNEC (all p < 0.05). Surgery was significantly associated with a favorable prognosis for LCNEC patients (p < 0.05). Primary site was not a prognostic factor for LCNEC (p > 0.05).

## Discussion

Through a large population-based cohort, we found that patients with pulmonary LCNEC who underwent surgery had significantly improved survival outcomes compared to non-surgically-treated patients. Moreover, patients who underwent lobectomy had the best outcome, followed by those who underwent partial/wedge/segmental resection. Thus, surgical resection should be considered in the treatment of stage I-Ⅲ LCNEC. As previously mentioned, pulmonary LCNEC shares some similar characteristics with SCLC, and chemotherapy and radiotherapy form the current standard treatment for patients with SCLC 26. Consequently, we explored the role of radiotherapy in the treatment of LCNEC patients. We found that radiotherapy significantly improved survival time only in stage Ⅲ LCNEC. Subsequently, we divided patients into two groups (surgery and no surgery). Patients in the no surgery group had better outcomes when undergoing radiotherapy. On the contrary, there was no survival benefit for LCNEC patients who underwent both surgery and radiotherapy. Meanwhile, compared to patients who underwent postoperative radiotherapy, patients with surgery alone had a longer survival time. Based on these results, radiotherapy could be applicable for patients unsuitable for surgery, especially for those with stage Ⅲ LCNEC. However, for patients undergoing surgery, combination treatment with radiotherapy should be carefully considered. The multivariable analysis demonstrated that survival depended on age, gender, surgery, radiotherapy and TNM stage.

Recently, several studies support surgical resection for patients with early-stage LCNEC. For example, Zacharias *et al*. 16 found that patients treated *via* complete resection after systematic nodal dissection had longer survival time than previously described. Grand *et al*. 17 reported that surgical resection improved survival in about one third of patients. Roesel *et al*. 15 found that surgical treatment can achieve satisfactory results in early stages pulmonary LCNEC. The overall 1-, 3- and 5-year survival rates for the surgery group were 83.7%, 63.2%, and 53.8%, respectively. Our findings are in agreement with these retrospective studies. Data on effectiveness of radiotherapy in pulmonary LCNEC are limited. Rieber *et al*. 23 conducted a retrospective analysis to investigate treatment outcome following multimodal treatment in 70 patients with LCNEC. In patients with incompletely resection and postoperative radiotherapy, 2- and 5-year survival rates were 50 and 30%, respectively. The authors concluded that the administration of radiotherapy contributes to acceptable results of multimodal treatment regimes. Prelaj *et al*. 24 showed that patients undergoing thoracic radiotherapy had higher mPFS and mOS (12.5 *vs*. 5 months, p = 0.02 and 28.3 *vs*. 5 months, p = 0.004). Nevertheless, further research is needed to assess the effectiveness of radiotherapy in patients with pulmonary LCNEC.

In this study, data on chemotherapy were not accessed. However, many authors support the use of chemotherapy in LCNEC patients. In a prospective study, Iyoda *et al*. 21 observed that adjuvant chemotherapy, consisting of cisplatin and VP-16, administered post-surgery, improved the prognosis of LCNEC patients. Christopoulos *et al.* 19 conducted a multicenter phase Ⅱ trial, and found that everolimus in combination with carboplatin and paclitaxel was an effective first-line treatment for patients with stage Ⅳ LCNEC. Derks *et al*. 27 thought that NSCLC-t chemotherapy (median survival: 8.5 months) resulted in a better prognosis compared to NSCLC-pt (median survival: 5.9 months) and SCLC-t (median survival: 6.7 months) chemotherapy. These studies showed that chemotherapy is paramount for effective treatment.

The present retrospective study has some limitations. On the one hand, several clinical data were not available in the SEER database. For example, low grade (G1/G2) "LCNEC" and carcinoma with unknown Grading were not excluded, due to the limitation of SEER database. Specifically, we were unable to obtain chemotherapy data, which is important for prognosis evaluation. In addition, information about scope and dose of radiation was also lacking. Lastly, we could not evaluate positive or negative surgical margins. On the other hand, inherent selection bias is inevitable in a retrospective study.

## Conclusions

LCNEC is an aggressive and rare tumor, with generally poor prognosis. Nevertheless, our results indicate that Lobectomy seems to be the optimal treatment for patients with LCNEC in the early stages (I-II). For stage III LCNEC patients, radiotherapy could potentially have a positive effect on survival time, especially for patients unsuitable for surgical resection. However, we do not recommend the use of radiotherapy in patients undergoing surgery, based on our results, it may reduce survival time.

## Figures and Tables

**Table 1:** Characteristics of Patients with Stage I to III Large-Cell Neuroendocrine Carcinoma (n=1,619)

Characteristics	No. (%)
**Age(years)**	
<65	633(39.1)
≥65	986(60.9)
**Gender**	
Female	770(47.6)
Male	849(52.4)
**Race**	
White	1381(85.3)
Black	177(10.9)
Other	61(3.8)
**Marital status**	
Married	1387(85.7)
Unmarried	173(10.7)
Unknown	59(3.6)
**CHSDA region**	
East	855(52.8)
Nothern Plains	154(9.5)
Pacific Coast	566(35.0)
Southwest	44(2.7)
**Primary site**	
Upper lobe	1005(62.1)
Middle lobe	92(5.7)
Lower lobe	442(27.3)
Main bronchus	24(1.5)
Other	56(3.4)
**Surgery**	
No surgery	435(26.9)
Lobectomy	850(52.5)
Partial/wedge/segmental resection	278(17.2)
Pneumonectomy	56(3.4)
**Radiotherapy**	
Yes	1387(85.7)
No	232(14.3)
**TNM stage**	
Stage Ⅰ	869(53.7)
Stage Ⅱ	203(12.5)
Stage Ⅲ	547(33.8)
**Grade**	
Well differentiated; Grade I	8(0.5)
Moderately differentiated; Grade II	37(2.3)
Poorly differentiated; Grade III	771(47.6)
Undifferentiated; anaplastic; Grade IV	230(14.2)
Unknown	573(35.4)
**No. of nodes examined in surgery (surgery n=1,184)**	
0	132(11.1)
1-3	206(17.4)
4-10	429(36.2)
10+	286(24.2)
Unknown	131(11.1)
**No. of nodes positive in surgery (surgery n=1,184)**	
0	772(65.2)
1-3	211(17.8)
4-10	40(3.4)
10+	6(0.5)
Unknown	155(13.1)

**Table 2:** Univariate Analysis for Large-Cell Neuroendocrine Carcinoma Using Cox Proportional Hazards Model (n = 1,619)

	OS		LCSS	
Variable	HR(95%CI)	P value	HR(95%CI)	P value
**Age(years)**				
<65				
≥65	1.552(1.359-1.773)	<0.001	1.458(1.251-1.699)	<0.001
**Gender**				
Female				
Male	1.210(1.067-1.372)	0.003	1.213(1.048-1.403)	0.010
**Race**				
White				
Black	0.991(0.807-1.217)	0.930	0.969(0.762-1.231)	0.795
Other	1.015(0.736-1.400)	0.926	0.907(0.613-1.343)	0.625
**Marital status**				
Married				
Unmarried	1.076(0.875-1.323)	0.487	1.041(0.817-1.327)	0.746
Unknown	1.036(0.728-1.475)	0.845	0.976(0.644-1.481)	0.911
**CHSDA region**				
East				
Nothern Plains	1.008(0.807-1.259)	0.944	0.953(0.734-1.237)	0.716
Pacific Coast	1.048(0.915-1.201)	0.496	0.995(0.848-1.166)	0.947
Southwest	1.318(0.912-1.905)	0.142	1.494(1.004-2.223)	0.048
**Primary site**				
Upper lobe				
Middle lobe	1.155(0.894-1.493)	0.271	1.139(0.843-1.540)	0.396
Lower lobe	1.124(0.973-1.297)	0.111	1.135(0.962-1.339)	0.135
Main bronchus	1.764(1.141-2.727)	0.011	1.832(1.112-3.019)	0.017
Other	1.492(1.074-2.072)	0.017	1.283(0.851-1.937)	0.234
**Surgery**				
No surgery				
Lobectomy	0.334(0.289-0.386)	<0.001	0.279(0.236-0.329)	<0.001
Partial/wedge/segmental resection	0.468(0.389-0.564)	<0.001	0.416(0.336-0.515)	<0.001
Pneumonectomy	0.593(0.427-0.823)	0.002	0.522(0.356-0.764)	0.001
**Radiotherapy**				
Yes				
No	1.009(0.848-1.200)	0.918	1.098(0.903-1.336)	0.349
**TNM stage**				
Stage Ⅰ				
Stage Ⅱ	1.628(1.337-1.982)	<0.001	1.872(1.491-2.351)	<0.001
Stage Ⅲ	2.600(2.268-2.980)	<0.001	3.054(2.604-3.581)	<0.001
**Grade**				
Well differentiated; Grade I				
Moderately differentiated; Grade II	1.219(0.421-3.525)	0.715	1.171(0.341-4.021)	0.802
Poorly differentiated; Grade III	1.232(0.460-3.297)	0.678	1.221(0.392-3.805)	0.731
Undifferentiated; anaplastic; Grade IV	1.276(0.472-3.451)	0.631	1.271(0.403-4.012)	0.682
Unknown	1.668(0.623-4.466)	0.309	1.684(0.540-5.253)	0.369

**Table 3:** Multivariate Analysis for Large-Cell Neuroendocrine Carcinoma Using Cox Proportional Hazards Model (n=1,619)

Variable	Overall survival		Lung cancer-specific survival	
	HR (95% CI)	P value	HR (95% CI)	P value
**Age(years)**				
<65				
≥65	1.564 (1.365-1.793)	<0.001	1.491 (1.273-1.745)	<0.001
**Gender**				
Female				
Male	1.199 (1.056-1.362)	0.005	1.212 (1.046-1.406)	0.011
**Primary site**				
Upper lobe				
Middle lobe	1.211 (0.936-1.567)	0.145	1.216 (0.899-1.645)	0.205
Lower lobe	1.065 (0.920-1.233)	0.397	1.091 (0.921-1.293)	0.311
Main bronchus	1.120 (0.717-1.750)	0.619	1.069 (0.642-1.779)	0.799
Other	0.797 (0.568-1.118)	0.189	0.633 (0.416-0.965)	0.033
**Surgery**				
No surgery				
Lobectomy	0.464 (0.389-0.554)	<0.001	0.394 (0.321-0.482)	<0.001
Partial/wedge/segmental resection	0.648 (0.524-0.802)	<0.001	0.594 (0.465-0.758)	<0.001
Pneumonectomy	0.627 (0.446-0.880)	0.007	0.535 (0.361-0.795)	0.002
**Radiotherapy**				
Yes				
No	0.765 (0.637-0.920)	0.004	0.809 (0.658-0.995)	0.045
**TNM stage**				
Stage Ⅰ				
Stage Ⅱ	1.839 (1.499-2.256)	<0.001	2.099 (1.657-2.657)	<0.001
Stage Ⅲ	2.060 (1.735-2.444)	<0.001	2.238 (1.833-2.733)	<0.001

## Abbreviations

AJCC: American Joint Committee on Cancer
LCNEC: large-cell neuroendocrine carcinoma
LCSS: lung cancer-specific survival
NSCLC: non-small-cell lung cancer
NCI: National Cancer Institute
OS: overall survival
SCLC: small-cell lung cancer
SEER: Surveillance, Epidemiology, and End Results
IRB: Institutional Review Board
TNM: tumor-node-metastasis
